# Actinomycin D and Telmisartan Combination Targets Lung Cancer Stem Cells Through the Wnt/Beta Catenin Pathway

**DOI:** 10.1038/s41598-019-54266-z

**Published:** 2019-12-03

**Authors:** Ryan Green, Mark Howell, Roukiah Khalil, Rajesh Nair, Jiyu Yan, Elspeth Foran, Sandhyabanu Katiri, Jit Banerjee, Mandip Singh, Srinivas Bharadwaj, Shyam S. Mohapatra, Subhra Mohapatra

**Affiliations:** 1 Department of Molecular Medicine, University of South Florida, Tampa, FL 33612 USA; 2 Department of Internal Medicine, University of South Florida, Tampa, FL 33612 USA; 3 Center for Research and Education in Nanobioengineering, Morsani College of Medicine, University of South Florida, Tampa, FL 33612 USA; 4 James A Haley VA Hospital, Tampa, FL 33612 USA; 5 Transgenex Nanobiotech Inc., Tampa, FL 33613 USA; 6 College of Pharmacy & Pharmaceutical Sciences, Florida A&M University, Tallahassee, FL 32307 USA

**Keywords:** Cancer stem cells, Cancer therapy

## Abstract

The failure of lung cancer treatments has been attributed mostly to the development of drug resistance, however the underlying cellular and molecular mechanisms are poorly understood. Cancer initiating stem cells (CSCs), present in tumors in a small percentage, play critical roles in the development of drug resistance, metastasis, and cancer relapse. Hence, novel treatments targeting both bulk cancer cells and CSCs are under intense investigation. Herein, we report that lung cancer cells grown on a 3D fibrous scaffold form tumoroids that resemble *in vivo* tumors, expand CSCs, and provide a platform to identify anti-CSC drugs. The screening of an NCI library of FDA-approved drugs using tumoroid cultures led to identification of Actinomycin D (AD) as a top CSC inhibitor. Since CSCs are mostly resident in the tumor’s inner core, AD was combined with an angiotensin receptor antagonist, Telmisartan (TS), which is known to increase drug permeability in tumors and was shown to have anti-CSC activity. Our results showed that AD + TS administered intra-tumorally was significantly more effective than either drug alone in both syngeneic and xenograft mouse models. The results of mechanistic studies revealed that CSC expansion in tumoroids was associated with activation of β catenin signaling and that AD + TS treatment reduced active β catenin levels in tumors. Together, these results establish the utility of the tumoroid culture system to expand CSCs *ex vivo* for targeted drug screening, to identify promising novel treatments with both anti-CSC and anti-cancer effects, and to individualize treatments for metastatic drug resistant lung cancer patients.

## Introduction

Lung cancer remains the most deadly cancer of any type with an estimated 234,030 new lung cancer cases and over 154,000 deaths in 2018 in the U.S. alone^1^. Despite a myriad of FDA-approved treatment options, the 5 year survival rate for lung cancer is very low at 18.6%^2^, which is primarily due to the development of drug resistance in metastatic tumors^2,3^. There is increasing evidence in support of the hypothesis that a small fractional sub-population of cancer-initiating stems cells (CSCs) play a pivotal role in cancer evolution and drug resistance^4,5^. Current radio-, chemo-, and immune-therapies are not capable of eradicating CSCs, which could account for their lack of effectiveness in metastatic drug resistant cancers^6,7^.

The field of CSC research has been confronted with two major barriers. First, cell surface markers as well as functional assays used to identify CSCs vary for specific cancer types, thus a universal CSC marker is lacking^8^. Hence, CSCs are currently identified by their ability to initiate xenograft tumors in mice at very low dilutions^9^ and this assay is limited by lack of immune competence and genetic background-related heterogeneity. Second, the mechanism by which CSCs escape drug and radiation therapies is poorly understood with results differing according to cancer type or model system^7^. The intrinsic drug resistance mechanisms of CSCs may involve stemness and pro-survival genes in combination with their oncogenic driver mutations^10^, quiescence^11^, drug export^12^ and decreases in reactive oxygen species (ROS) in the tumor microenvironment^13^. Irrespective of the mechanism, treatments targeting CSCs remain a highly sought after goal and many strategies are under investigation including those targeting: (i) CSC developmental pathways such as Wnt, Notch, and hedgehog; (ii) their oxidative and glycolytic metabolism, and stromal components such as cancer associated fibroblasts which release TGFβ to stimulate CSC growth^5,14^. None of these has translated into a successful clinical treatment.

Several approaches are under investigation to expand CSCs in an effort to overcome the limitations inherent to their rarity in culture. These include (i) CSC expansion by successive rounds of growth in mouse, which is limited by strain variations, high cost, and time intensive protocols^15,16^; (ii) transformation of CSCs with reprogramming transcription factors such as Oct-4, c-myc and Sox-2^16^; (iii) culturing CSCs on low attachment plates with growth factors such as EGF and FGF; and (iv) expansion of CSCs in organoid cultures by mimicking the 3D tumor microenvironment^17,18^ which maximizes cell-matrix and cell-cell interactions and activates CSC signaling pathways^19^. To this end, we reported a polymeric fiber scaffold culture system that causes cancer cells to undergo EMT and facilitates CSC expansion with ease^20^. Screening of compounds using these tumoroid cultures led to identification of one lead compound, Actinomycin D (AD), a chemotherapeutic drug, which binds to DNA blocking the progress of RNA polymerases during transcription and inhibiting topoisomerases causing strain on the DNA molecule leading to double stranded breaks^21,22^. AD was shown to block expansion of CSCs in scaffold cultures.

Though AD is currently FDA-approved for anti-cancer therapy, its use is limited because it causes systemic toxic side effects. We reasoned that AD’s anti-CSC and anti-cancer activity may be better exploited by using a low dose of AD and intra-tumoral delivery in conjunction with other drugs. Since CSCs are mostly resident in the hypoxic inner core of the tumors, we selected the angiotensin receptor blocker telmisartan (TS) as it is known to increase the penetration of drugs into tumors via reduction in collagen production^23,24^. Also, TS was shown to decrease cancer cell proliferation through inhibition of PI3K signaling and activation of peroxisome proliferator activated receptor-γ (PPARγ)^25–27^. Thus, we tested the hypothesis that combination therapy involving AD and TS might increase anti-cancer and anti-CSC effects providing total cancer care. Our data shows that a combination of AD and TS synergistically inhibited both tumor growth and expansion of CSCs *in vitro* and *in vivo*, and this inhibition is mediated by decreased signaling through Wnt/β-catenin pathway.

## Results

### 3D tumoroid culture promotes CSC growth in lung cancer cell lines

**Figure 1:**
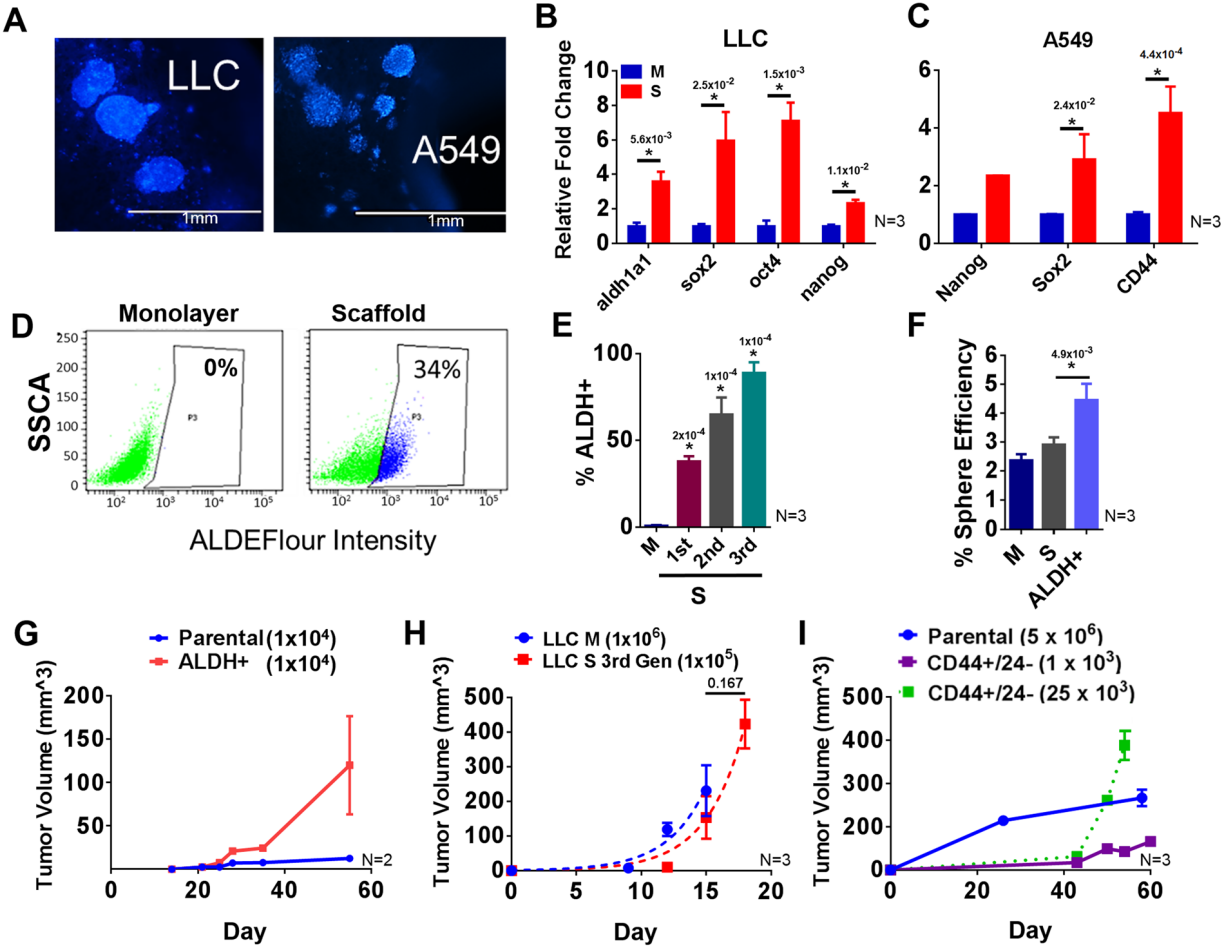
(**A**) Fluorescence micrographs of lung cancer cell lines (LLC1 and A549) cultured on 3D scaffold. Cells (5,000/well) were seeded onto scaffold matrix in 96 well plates and NucBlue stained cells visualized by fluorescent microscopy on day 6. (**B–F**) Cells were cultured on scaffold as described in (**A**). On day 6, RNA was isolated from cell pellets, and CSC marker gene expression in monolayer vs 3D scaffold cultures was assayed by qPCR. ANOVA (Dunnett) N = 3, *p ≤ 0.05. LLC1 (**B**) and A549 (**C**). ALDH enzyme activity using the ALDE Fluor kit (Stem Cell Technologies) (D). Average ALDH activity in LLC1 cells cultured on 3D scaffold over multiple 6 day generations. ANOVA (Dunnett) N = 3, *p ≤ 0.05 (**E**). ALDH high expressing LLC1 cells were isolated from the parent scaffold population by FACS (BD FACS Aria) and cultured in low attachment conditions at a concentration of 2 cells/µL media for 6 days. Sphere efficiency is shown. One way ANOVA (Tukey) was used to calculate significance. N = 3, *p ≤ 0.05 (**F**). (**G–I)** Tumor initiation ability of CSC population isolated from LLC1 or A549 cells. ALDH high LLC cells were injected subcutaneously into both flanks of C57/BL6 mice using 10,000 cells per flank and the size of the resulting tumors was measured by caliper (N = 2) (**G**). 100,000 LLC1 cells obtained from 3^rd^ generation tumoroid culture were injected subcutaneously into both flanks of C57/BL6 mice and tumor growth was compared to 1 million LLC1 cells obtained from monolayer culture. The best fit growth curves for monolayer and 3^rd^ generation tumoroid derived tumors were determined using the exponential growth equation Y = Y0 * exp(k * X). The growth rate constants for each curve (**K**) were compared using the Extra sum of squares F test. N = 3 (**H**). CD44+/24− A549 cells were injected subcutaneously into the flanks of Nod/SCIID immunocompromised mice using the cell numbers indicated for each group (N = 3) (**I**). The number above each star represents the p value obtained for that comparison.

When cancer cells (mouse Lewis Lung Carcinoma 1 (LLC1), human A549) were cultured on 3D scaffold they formed tumor-like organoids, referred to as tumoroids (Figs. 1A and S1). After six days of culture, the tumoroids grew to a diameter of 50–200 µM. In order to evaluate whether cells cultured on the 3D scaffold are enriched in CSCs, we examined stemness by ALDH activity and expression of the classical pluripotency related stem cell transcription factors Sox2, Oct4, and Nanog (Fig. 1B,C). The expression of aldh1a1, sox2, oct4, and nanog increased in LLC1 cells cultured on 3D scaffold while Sox2, Nanog, and CD44 expression was increased in A549 cells (Fig. 1B,C). Recently, other groups have highlighted the role of the Lgr5 gene/protein in lung CSCs and we have also found Lgr5 expression to be increased in the 3D tumoroid cultures (Fig. S2)^28^. Culturing LLC1 as tumoroids on 3D scaffold increased their ALDH activity (Fig. 1D). Each successive passage represents 6 days of culture and after 3 passages (18 days) ALDH activity was increased from 0.3% to 87.5% (P = <0.0001) (Fig. 1E). This suggests that LLC1 cultured on scaffold are becoming more stem-like. Fluorescent activated cell sorting (FACS) isolated ALDH high LLC1 were assayed for attachment independent growth using a sphere forming assay and compared to LLC1 monolayer and bulk LLC1 collected from scaffold. The ALDH high population was found to have double the sphere forming efficiency (4.8%) when compared to monolayer (2.3%) or bulk scaffold (2.8%) (Fig. 1F). This increased sphere forming ability and resistance to anoikis further supports the conclusion that culture on 3D scaffold is able to enrich CSCs. Further, we isolated these ALDH high LLC1 cells and tested for their ability to initiate tumors *in vivo*. The ALDH high cells, isolated using FACS when injected subcutaneously into the flanks of C57BL/6 mice, formed tumors at a density of 10,000 cells per flank. The ALDH high LLC1 cells were able to develop larger tumors at a faster rate than the bulk cells collected from scaffold (Fig. 1G). Similarly, we tested the tumor initiation ability of 3^rd^ generation LLC1 tumoroids, which we have shown to exhibit increased ALDH activity, compared to LLC1 cells from monolayer culture (Fig. 1H). This data suggests that the ALDH high cells have a nearly ten times greater tumor initiation ability than the ALDH low cells while growth rates of tumors did not differ significantly between both groups. This increased tumor initiating ability confirmed that ALDH high LLC1 are indeed CSCs and the fact that ALDH increases as a result of scaffold culture means that scaffold culture does enrich CSCs. Similarly, CD44+/24− A549 population isolated by magnetic separation (MACS) was assayed for their *in vivo* tumor initiation ability compared to their parental un-sorted A549 by subcutaneous injection into the flanks of Nod/SCID immunocompromised mice at the indicated concentrations (Fig. 1I). Tumors initiated with CD44+/24− A549 were found to grow more rapidly even when initiated with fewer cells than parental A549.

### CSC can be maintained in 3D culture with increased expression of stemness genes

**Figure 2:**
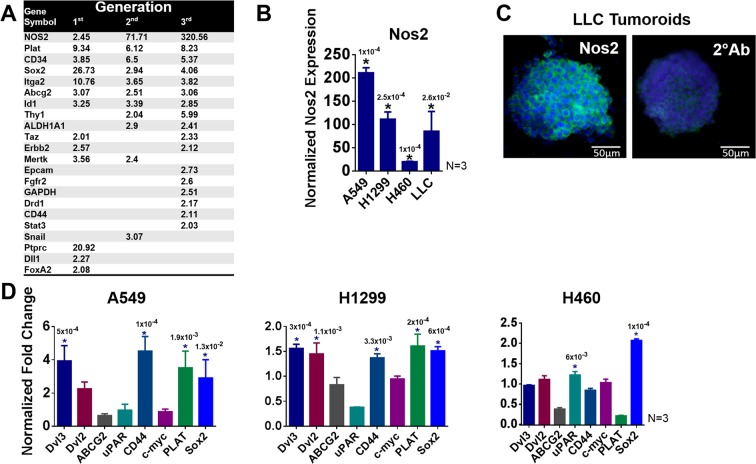
(**A)** A qPCR array was used to identify changes in RNA expression for CSC related genes in first, second, and third generation LLC1 scaffold culture. Data was normalized to LLC1 monolayer and fold change values are presented as a table. (**B**,**D**) Nos2 (**B**) and CSC related gene expression in H1299, A549, H460 and LLC1 cultured on scaffold for 6 days normalized to monolayer by qPCR (**D**). Data represents increase in gene expression in tumoroids as compared to monolayer. One Way ANOVA (Dunnett) was used to calculate significance (Prism) N = 3, *p ≤ 0.05. (**C)** ICC for Nos2 protein in 1st Gen LLC1 tumoroids fixed on day 6 of culture (600X magnification). The number above each star represents the p value obtained for that comparison.

A qPCR array (Qiagen) was used to identify gene expression changes in LLC1 tumoroid culture through multiple 6 day passages. A scatterplot of overall gene expression changes occurring between LLC1 monolayer culture and 3^rd^ generation scaffold is also provided (Fig. S3). Several genes were found to be up-regulated including Nos2, PLAT, and CD34 many of which are involved in Wnt/β catenin signaling, a pathway known to drive cell proliferation and regulate cell-cell adhesion in cancer (Fig. 2A)^29–32^. Nos2 showed the greatest increase in scaffold culture and this result was replicated by qPCR in the human non-small cell lung cancer (NSCLC) cell lines A549, H1299, and H460 as well as in LLC1 when cultured on scaffold (Fig. 2B).

Immunocytochemistry (ICC) for the Nos2 protein was used to confirm its expression as seen in the qPCR array (Fig. 2C). Nos2 (stained in green) is highly expressed in LLC1 tumoroids. Many of the other stemness genes shown to increase in LLC1 cultured on scaffold were also increased in human NSCLCs cultured on scaffold for 6 days albeit at a somewhat lower fold change (Fig. 2D). Together, these results demonstrate that the tumoroid culture can promote stemness in human as well as mouse NSCLC, and the expression of these genes varies depending upon the cell line.

### AD treatment reduces CSC characteristics *In Vitro*

**Figure 3:**
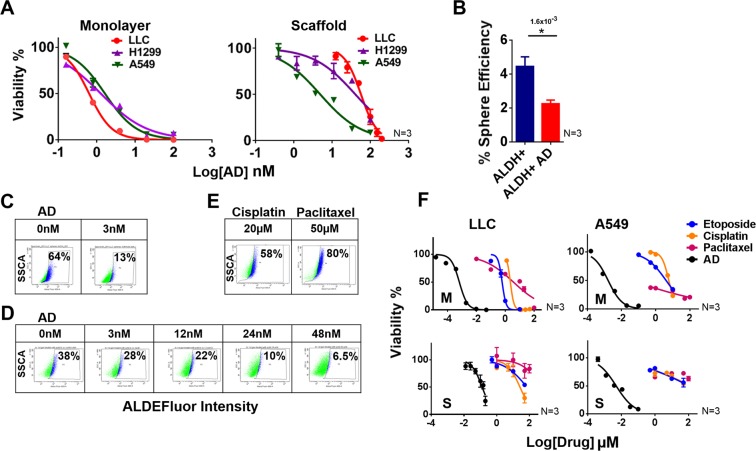
(**A)** Dose response curves for mouse and human lung cancer cell lines cultured on monolayer vs 3D scaffold. Cells were plated on scaffold in 96 well plates at a density of 5,000 cells per well. Serial dilutions of AD were added on day 4 and cell viability was assayed on day 6 by CellTiter-Glo. (Promega) N = 3. (**B)** Sphere formation efficiency of sorted ALDH high LLC1 cells with or without AD treatment (0.5 nM) Student’s t-test N = 3, *p ≤ 0.05. (**C,D)**. ALDH activity of LLC1 cells cultured as spheres on low attachment plates with or without 3 nM AD treatment (**C**) or as tumoroids on 3D scaffold after treatment with escalating doses of AD (**D**), as assayed by flow cytometry using ALDE Fluor kit. (**E)** ALDH activity of LLC1 cells cultured as tumoroids was assayed after 48 hr treatment with cisplatin (20 µM) or paclitaxel (50 µM). (**F)** Dose response curves for the chemotherapeutic drugs etoposide, cisplatin, and paclitaxel compared with AD in the LLC1 and A549 cell lines cultured either as monolayer (M) or on 3D scaffold (S) N = 3. The number above each star represents the p value obtained for that comparison.

Toward repurposing drugs for CSC inhibition, we initially screened an NCI Diversity library of FDA-approved drugs using H460 human lung cancer cell line which contains mutations in the clinically relevant genes EGFR and KRAS (Fig. S4). We found that AD was one of the top candidates, significantly inhibiting growth of lung cancer cells on monolayer in nanomolar concentration with IC_50_ values of 0.63 nM (LLC1), 1.28 nM (H1299), and 1.64 nM (A549) (Fig. 3A). Tumoroid culture was found to slightly raise the IC_50_ of AD in cell lines, 127 nM (LLC1), 40 nM (H1299), and 5 nM (A549) (Fig. 3A). Also, AD reduced the sphere forming efficiency of LLC1, an indicator of stemness, in isolated ALDH high cells from 4.8% in untreated cells down to 2.2% with only a 0.5 nM AD treatment at the start of sphere culture (Fig. 3B). Since CSC marker ALDH was found to be highly active in LLC1 cells cultured under low attachment conditions, we tested the effect of AD in a tumorsphere assay, and indeed, treatment with as little as 3 nM AD was able to reduce ALDH activity in tumorspheres from 64% to 13% (Fig. 3C). LLC1 cells cultured as tumoroids on scaffold were also treated with AD in increasing concentrations (3–48 nM) and a dose-dependent reduction in ALDH activity was observed (Fig. 3D). In contrast, when LLC1 tumoroids were treated with the clinically used chemotherapy drugs cisplatin or paclitaxel, an increase in the ALDH activity was observed (Fig. 3E). Furthermore, while the drugs etoposide, cisplatin, and paclitaxel were found to successfully inhibit the growth of LLC1 (IC_50_ = 0.6 µM, 2.3 µM, 3.7 µM) and A549 (IC_50_ = 4.1 µM, 5.8 µM, 0.04 µM) monolayer cultures these same drugs had much higher IC_50_ values in LLC1 (IC_50_ = 69 µM, 26 µM, 514 µM) and A549 (IC_50_ = 106 µM, 146 µM, >500 µM) scaffold based tumoroid cultures while AD remained potent in both monolayer and scaffold (Fig. 3F). These results suggest that AD can decrease the stem-like properties of cancer cells while cisplatin and paclitaxel do not.

### AD + TS treatment synergistically reduces *In Vitro* Cell viability and decreases stemness

**Figure 4:**
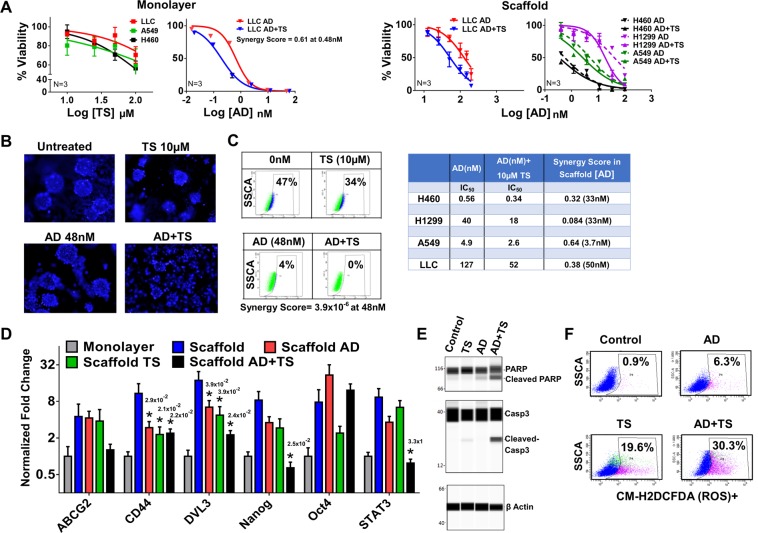
**(A)** IC_50_ values were determined for cell lines cultured as tumoroids on scaffold treated with varying concentrations of AD in the presence or absence of TS (10 µM). Drugs were added on day 4 of culture and viability was assayed on day 6 using CellTiter-Glo. Combination index was calculated using CompuSyn Software by comparing the effects of AD and TS alone vs in combination. Dose response to TS alone in monolayer cultures is shown along with the comparison between AD and AD + TS treatment response in LLC1 monolayer culture. IC_50_ and combination index values are provided. (**B)** LLC1 cells were cultured as tumoroids on scaffold. On day 4 wells were treated with either AD, TS, or both in combination. On day 6 cells on scaffold were stained with NucBlue dye and examined by fluorescent microscopy. (**C)** LLC1 cells cultured and treated as in (**B**) were collected on day 6 of culture and ALDH activity was assayed by ALDE Fluor kit. (**D,E)** LLC1 cells were cultured and treated as in (**A**) and as monolayer culture. Cells were treated on day 4 and collected on day 6 (48 hr treatment), and expression of CSC related genes were assayed by qPCR. ANOVA(Dunnett) N = 3, *p ≤ 0.05 (**D**). Expression of PARP and Caspase3 (both full length and cleaved) was assayed using Wes (**E**). (**F)** Production of ROS was assayed in LLC1 monolayer cultures treated with 0.4 nM AD, 1 µM TS, or the combination for 48 hr. CM-H2DCFDA staining was assessed using fluorescent microscopy (Fig. S11) (while brightfield images were also collected for each field as a reference) and by flow cytometry using the FITC channel. The number above each star represents the p value obtained for that comparison.

Because of AD’s potential toxicity, we tested the effect of combination therapy involving AD and TS in an effort to decrease the dose of AD necessary to achieve its anti-cancer and anti-CSC effects. LLC1-tumoroids were treated with a combination of AD at varying concentrations plus 10 µM TS (Fig. 4A). The addition of TS was found to reduce the IC_50_ of AD from 127 nM to 52 nM in a synergistic fashion (calculated using CompuSyn software) (Fig. S5). This effect was also seen in tumoroid cultures of human lung cancer cell lines (Fig. 4A); however, the largest combination effect was seen in LLC1 tumoroids after third passage revealing the treatment’s ability to target CSC (Fig. S6). The IC_50_ of TS in LLC1 was found to be 278 µM, in H460 125 µM, and in A549 304 µM indicating that TS alone is not a potent inhibitor of cancer cell growth (Fig. 4A). The AD + TS combination was also shown to be effective in LLC1 monolayer culture with the addition of 5 µM TS reducing the IC_50_ of AD to 0.19 nM from 0.63 nM for AD alone. Further, the combination treatment of AD plus TS was found to change the morphology of LLC1 tumoroids, exhibiting more disaggregation of tumoroids (Fig. 4B) compared to tumoroids treated with AD or TS alone. We also assayed these tumoroid cultures for ALDH activity and while AD greatly reduced ALDH activity, AD + TS treatment completely abrogated ALDH activity in a synergistic fashion (Fig. 4C). We assayed the effect of AD + TS on CSC-related gene expression changes by qPCR following a 48 hr drug treatment. LLC1 tumoroids treated with AD or AD + TS were found to have reduced expression of several stemness related genes including abcg2, dvl3, nos2, and plat (Fig. 4D), but not Oct4. TS treatment alone reduced expression of CD44, Oct4, and Nanog revealing that it may have broader anti-CSC effects than simply increasing the uptake of AD. We also checked the expression levels of these same genes after a 24 hr treatment and found a pattern similar to the 48 hr results albeit with somewhat smaller magnitude of difference between groups (Fig. S7). Furthermore, we tested the effect of AD + TS treatment on Wnt/stemness genes in 3^rd^ generation LLC1 tumoroids and found a slightly different pattern of expression with ABCG2, CD44, and Lgr5 showing the largest reduction. This finding highlights the complexity of the Wnt signaling pathway and future studies will be needed to reveal the exact regulatory mechanisms controlling expression of specific Wnt target genes in the tumoroid model (Fig. S8).

An Ingenuity Pathway Analysis (IPA, QIAGEN Inc.) was performed to predict mechanisms of interaction between AD and TS based on published literature (Fig. S9), which suggested that caspase 3 could directly interact with both AD and TS^33^. To test this, we performed western immunoassays for the levels of PARP and caspase3 to measure effects of AD + TS on apoptosis (Figs. 4E, S10). Following AD + TS treatment levels of both cleaved PARP and cleaved caspase3 were found to increase indicating that LLC1 tumoroids were undergoing apoptosis. The IPA analysis also predicted AD and TS to interact via generation of ROS. To test this, we used a ROS indicator dye, CM-H2DCFDA, to determine whether ROS production is increased in LLC1 following treatment (Fig. S11). Figure 4F shows that TS is a potent activator of ROS resulting in a 19% increase after 1 µM treatment. When combined with a low dose of AD (0.4 nM), which is only able to generate a 5% increase on its own, the AD + TS combination induced a greater increase in ROS than either drug alone at almost 30%.

### AD + TS treatment reduces tumor growth *In Vivo*

**Figure 5:**
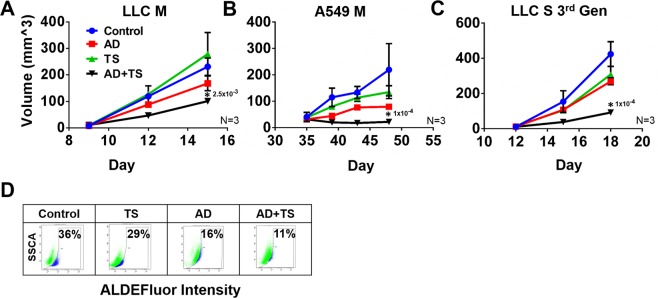
**(A–C)** *In vivo* tumor treatment. Cells were injected subcutaneously into the flanks of mice, 1 million LLC1 monolayer cells into C57BL/6 (**A**), 5 million A549 monolayer cells into Nod/SCIID mice (**B**) or 100,000 LLC1 3^rd^ generation tumoroids into C57BL/6 (**C**). Mice were treated with 50 µg/kg AD, 1 mg/kg TS, or combination by intratumoral injection every 3 days starting when tumors reached 3 mm in diameter. Tumor growth was monitored by caliper measurement and mice were sacrificed when tumors reached 10 mm in diameter. For all experiments N = 3 control, 4 AD, 4 TS, 7 combination. ANOVA (Tukey), *p ≤ 0.05. (**D)** Tumors initiated with LLC1 3^rd^ generation scaffold cells were assayed for ALDH activity using ALDE Fluor kit following treatment described in (**C**). The number above each star represents the p value obtained for that comparison.

We tested the effectiveness of intra-tumoral AD and AD + TS treatments in reducing tumor burden in LLC1, (10^6^ cells) injected, subcutaneous flank tumors in C57BL/6 mice. Mice were treated with AD (25 µg/kg or 50 µg/kg) starting on day 10 post injection, every 3 days for a total of 3 treatments and tumor diameters measured. The results showed that only the 50 µg/kg AD treatment was found to significantly reduce tumor size (Fig. S12). In order to determine the anti-tumor effect of TS this experiment was repeated with the addition of TS and AD + TS groups using only 50 µg/kg AD and 1 mg/kg TS. Both AD and the combination treatment reduced tumor size although the combination had a greater effect, while TS treatment did not reduce tumor size (Fig. 5A). We also tested the effectiveness of AD + TS treatment in a xenograft model using A549 subcutaneous flank tumors in Nod/SCIID mice, the results showed no significant effect of TS alone, slow tumor growth for AD alone and greatest reduction in tumor size in the AD + TS treated group (Fig. 5B). We then tested the effectiveness of AD and TS using 3^rd^ generation LLC1 tumoroids, which contain the highest percentage of drug resistant-CSCs. We injected 10^5^-CSC-rich third passage LLC1-tumoroid cells into flank tumors and treated these tumors as above. The results showed a significantly stronger treatment effect for the AD + TS group vs AD or TS alone groups (Fig. 5C). Importantly, we observed no significant differences in body weight change between any of the treatment groups indicating a lack of overt toxicity (Fig. S13) We assessed the stemness of these treated LLC1 tumor cells by assessing ALDH activity using flow cytometry. The TS, AD, and AD + TS treated tumors were all found to have reduced ALDH activity compared to the control group (Fig. 5D). This indicates that AD and AD + TS treatments are effective at reducing CSC in addition to reducing overall tumor size and burden.

### AD + TS treatment targets lung cancer CSC through the Wnt/β catenin pathway

**Figure 6:**
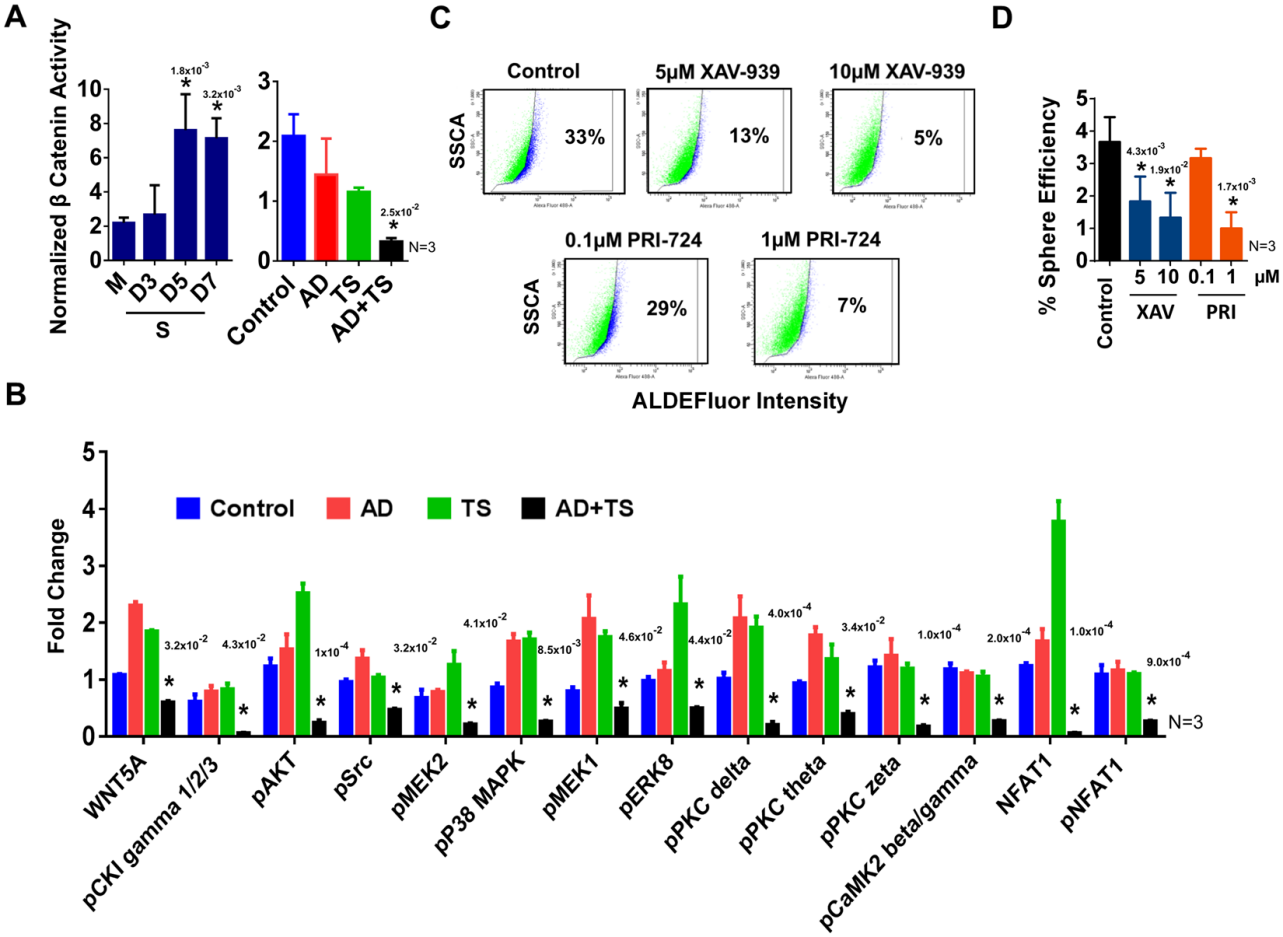
(**A)** LLC1 cells were cultured as monolayer or on scaffold. On day 2 of culture cells were transfected with a β catenin reporter plasmid and luminescence was assayed at the indicated timepoints (top panel). LLC1 monolayer were also transfected with β catenin reporter plasmid and treated with TS (1 µM), AD (0.3 nM) or combination after 24 hr (bottom panel). Luminescence was assayed at 72 hr. ANOVA (Dunnett) N = 3, *p ≤ 0.05. (**B)** Relative protein abundance and phosphorylation for Wnt pathway proteins in drug treated LLC1 tumors. Protein was collected from drug treated LLC1 tumors (Fig. 5C) and a phosphor-antibody array assay was performed to determine changes occurring in the Wnt pathway (Full Moon Biosystems). Cy-3 developed array chips were scanned, and images were analyzed using ImageJ to determine relative normalized staining intensity for each antibody. Fold change for select proteins is shown. ANOVA(Dunnett) N = 3, *p ≤ 0.05. (**C)** LLC1 cells were cultured on scaffold. On day 4 of culture cells were treated with the Wnt pathway inhibitors, PRI-724 or XAV-939 at the indicated concentrations. ALDH activity was assayed after 48 hr treatment. (**D)** Sphere formation efficiency was determined for LLC1 cells cultured and treated as in (**C)**. ANOVA (Dunnett) N = 3, *p ≤ 0.05. The number above each star represents the p value obtained for that comparison.

After verifying increased expression of several genes that are involved in or targets of β-catenin signaling in tumoroids including Dvl2, Dvl3, STAT3, CD44, Oct4, Sox2 by qPCR we also performed a TOPFLASH assay for activated β-catenin which showed that LLC1 tumoroids had higher levels of active β-catenin than LLC1 monolayer cultures and monolayer LLC1 cultures treated with AD + TS had significantly reduced levels of active β-catenin (Fig. 6A).To further investigate the effects of the combination treatment on the Wnt/β-catenin pathway we performed an analysis of protein phosphorylation on drug treated LLC1 tumor samples using an antibody array chip (Full Moon Biosystems). Overall activation of the Wnt pathway was reduced by the combination treatment as displayed by heatmap in Fig. S14 and several key changes to the pathway were observed including decreased production of Wnt 5a and decreased activation of CaMk2 and CKI gamma indicating that the treatment may be affecting the Wnt/calcium pathway in addition to the canonical Wnt/β-catenin pathway (Fig. 6B). Downstream effectors of the Wnt pathway were also found to be reduced following AD + TS treatment including phospho-MAPK, phospho-Src, and phospho-AKT, indicating that the processes of cell growth and proliferation controlled by these pathways are diminished by AD + TS treatment (Fig. 6B). To determine if the observed decrease in Wnt signaling could lead to reduced CSC properties, we treated LLC1 tumoroid cultures with two small molecule inhibitors of the Wnt pathway, PRI-724 and XAV-939. These function by blocking the association between β-catenin and its co-activator CREB binding protein (CBP) and by inhibiting the activity of Wnt intermediary tankyrase, respectively. After 48 hr treatment we observed a decrease in ALDH activity and sphere formation efficiency confirming that inhibition of Wnt signaling is able to reduce CSC properties in LLC1.

## Discussion

The results of our investigations presented herein focus on potential mechanisms of CSC expansion in tumors using *in vitro* and *ex vivo* tumoroid cultures, identification and evaluation of an FDA-approved drug using the tumoroid cultures and mouse models of lung cancer, formulation of a combination therapy with significant synergy for its anti-CSC and anti-cancer activities, and finally unravelling the mechanism underlying their synergy. The most notable findings from our studies which provide evidence and new insights to the field include: (i) data showing a combination of AD and TS synergistically inhibits tumor growth and CSC number and activity *in vitro* and *in vivo*, (ii) the results of transcriptomic analyses which identified the Wnt/β-catenin pathway as crucial for lung CSC development and re-confirmed the importance of known targets such as Sox2, ALDH, and Nos2.

The evidence that our 3D polymeric fiber scaffold-based tumoroid culture model represents a new approach to CSC enrichment through enhancement of cell-cell and cell-matrix interactions is significant in that this method avoids the artificial manipulations of transformation and growth factor stimulation. The *ex vivo* tumoroid cultures allow the study of CSCs in an *in vivo* like TME and permits expansion and isolation of CSCs so that their unique properties can be studied in interaction with the non-CSC cells. Our results demonstrating expansion of CSCs in lung cancer cell lines on our novel tumoroid culture model is consistent with other reports^34,35^, although unlike the current method they lack *in vivo* verification of stemness. Also, our tumoroid culture method of expanding CSCs is unique, easy to perform and does not require added growth factors. Further, this tumoroid platform was successfully used for screening therapies targeting CSCs identifying the AD + TS combination treatment, which was able to reduce tumor burden and stemness *in vitro* and *in vivo*. In addition, these results suggest that tumoroid cultures are able to maintain and expand the rare CSC populations over multiple passages enabling more in-depth studies of the molecular mechanisms responsible for CSC development in a variety of cancers.

A major finding of our investigation is the mechanism underlying enrichment and maintenance of lung cancer CSC in tumoroids derived from cancer cells on the unique polymeric nanofiber scaffold inspired tumoroid culture system. Herein, several molecular analytical methods helped identify specific markers and pathways critical for enrichment of CSCs. Transcriptomic analyses of tumoroids allowed us to study the gene expression changes involving the Wnt/β-catenin pathway being crucial for lung CSC development and re-confirmed the importance of known Wnt targets such as Sox2, CD44, ALDH, and Nos2. Although ALDH gene expression did show some variation in the tumoroid model in different experimental settings, to our best understanding, the measure of ALDH activity may be a more relevant CSC marker than the level of ALDH mRNA as increased expression does not always lead directly to increased activity of the enzyme. We have observed a consistent 30–50% increase in ALDH activity in 1st generation tumoroids as compared to monolayer culture throughout many independent experiments which supports the conclusion that CSC enrichment is promoted by tumoroid culture. Furthermore, because Nos2 is a Wnt target gene its increased expression in tumoroids provides some evidence that β-catenin activity is increased by scaffold culture. Nos2 is known to promote drug resistance in lung cancer by activation of the canonical Wnt pathway through inhibition of DKK1 and to promote the growth of CSCs in glioma via the Notch pathway^36,37^. In this way, Nos2 may be acting in a positive feedback loop to promote stemness in tumoroid cultures. The nitric oxide which is released by these CSCs may also play a role in increasing vascular permeability and inducing immune cell anergy, but this area requires further investigation.

Another major finding of these investigations is the demonstration of AD and TS as potent CSC inhibitors. The cyclic peptide antibiotic AD, first discovered in actinomyces soil bacteria, was shown to have anti-tumor activity and is FDA-approved to treat Wilms tumor, rhabdomyosarcoma and choriocarcinoma^38^. It initiates apoptosis in many cancer cell lines^22,39^ when administered in high doses through its DNA intercalating genotoxic effects. Further, in lower doses it has been used in cyclotherapy to protect healthy cells from the off-target effects of other chemotherapies by activating p53 and causing the non-cancerous cells to undergo cell cycle arrest. In addition, we have previously demonstrated that AD may have anti-CSC effects in breast cancer by reducing expression of Sox2^40^. TS, originally selected to increase the permeability of AD in tumors, was also found to possess anti-CSC activity which is a novel finding. TS is known to cause metabolic changes within tumors that increase fatty acid oxidation and reduce inflammation^26,27^. Though precise effects of TS on CSCs are unclear, the use of metabolism to target CSCs is a rapidly expanding area of research and TS’s role in this area warrants further investigation^41^. We have investigated the synergy of AD + TS in LLC tumoroid and monolayer culture and found that the combination acts synergistically in both models albeit with a higher CI in monolayer than in the tumoroid culture. This indicates that AD + TS can be effective in the low CSC bulk tumor as well as the CSC enriched tumoroid cultures. Nevertheless, the need to lower the dosage of AD and the observation of synergistic anti-CSC effects of AD and TS prompted investigation of their use for an intra-tumoral combination therapy in syngeneic and xenografted mice. We have demonstrated in these studies that AD + TS is able to reduce bulk tumor burden (Fig. 5A) possibly through the well-known mechanism of transcription inhibition by AD or by induction of apoptosis (Fig. 4E). We have also shown that AD + TS can target tumors enriched in CSCs (Fig. 5C) and this targeting is at least partially due to reduction in Wnt signaling (Fig. 6). While AD and TS alone did show some effect in reducing tumor burden, unfortunately these changes were not significant. This is likely due to the experimental variation inherent to *in vivo* experiments. Future experiments with larger N will be required to make strong conclusions about the effectiveness of TS or AD alone in in monolayer vs 3^rd^ generation tumors.

To the best of our knowledge such a combination of therapeutics targeting both cancer cells and CSCs has not been reported previously. The use of TS to enhance the effect of other drugs to treat lung cancer in mice has been reported previously both by oral and inhalation delivery^23,24^. AD is currently FDA approved for intravenous (IV) administration. We have used an intratumoral administration of AD in the present study in order to overcome the challenges of IV chemotherapeutic delivery in mice (repeated dosing, accuracy of injected volume, ulceration of the injection site); however, future studies will need to confirm the effectiveness of the treatment delivered by more clinically relevant routes such as oral TS plus IV AD.

**Figure 7:**
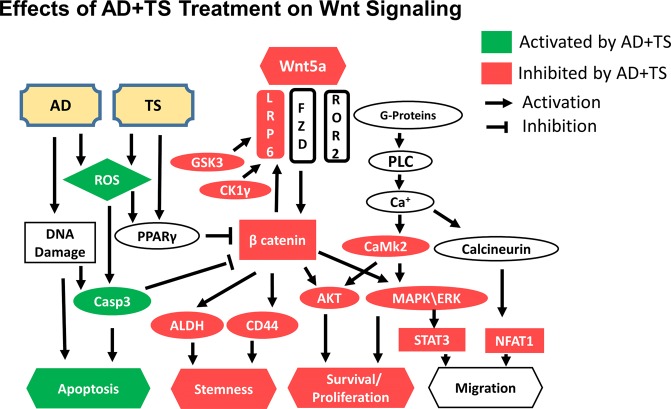
Summary of changes to the Wnt pathway as a result of AD + TS treatment.

Another significant finding of our studies is that the efficacy of the novel AD + TS combination treatment approach involves anti-CSC effects, which are at least partially mediated by alterations in the Wnt/β-catenin pathway, leading to decreased activity of key cell proliferation signals and ultimately apoptosis (Fig. 7). The finding that β-catenin activity plays a critical role in anti-CSC activity is significant because it is known to drive both metastasis and the CSC phenotype in cancers other than lung cancers such as breast cancer, colon cancer, and hepatocellular carcinoma and because there are currently no drugs approved to target the Wnt/β-catenin pathway in cancer^42,43^.

This finding is particularly relevant to NSCLC because Wnt/β-catenin signaling is commonly found to be elevated in this disease and the loss of endogenous regulators of Wnt/β-catenin such as DKK-3, AXIN, or APC have been associated with poor prognosis^44^. The Wnt ligand Wnt5a, which we have found to be elevated in LLC1 tumors and which was reduced by our combination treatment, was reported to act as either a tumor promotor or tumor suppressor depending on the cancer type^45^. It has previously been associated with tumor cell proliferation and expansion of CSCs in NSCLC^46^. This diverse activity may be attributed to the fact that Wnt5a can either activate or inhibit β-catenin phosphorylation through the canonical Wnt pathway depending on the cellular context, i.e., presence or absence of frizzled (FZD)^47^. Thus, in the presence of FZD, Wnt5a binds to ROR1/2 (receptor tyrosine kinase like orphan receptor), activates β-catenin after binding to the FZD-4/5 LRP5/6 complex, leading to β-catenin/TCF target gene transcription^48^ and suppression of downstream PPARγ activation. In this way, activation of canonical Wnt or Wnt/calcium signaling which lead to cell proliferation and acquisition of CSC traits can be linked to a decrease in PPARγ activity^49^.

Alternatively, when Wnt5a binds to ROR2 in the absence of FZD, β-catenin activation is inhibited^47^. TS is known to activate PPARγ as a partial agonist, which can in turn inhibit activation of β-catenin either by stabilizing the APC/GS3kβ destruction complex, activating the Wnt inhibitor DKK, or by inhibiting the translocation of β-catenin to the nucleus^50–52^. This mechanism has been shown to be involved in the differentiation of mesenchymal stem cells into osteoblasts^48^. Thus, our results implicate Wnt signaling as a target of this treatment and we report the treatment’s effects on both the canonical and Wnt/calcium pathways. Nonetheless, further investigation into the precise regulatory mechanism by which these drugs induce changes in the ROS and PPARγ activities and alter the transcription and function of Wnt-target genes to reduce the stemness of CSCs is needed.

Further, both AD and TS stimulate ROS production which in turn can enhance apoptosis^22,53^. This may play a key role in the treatment’s ability to specifically target CSCs through the Wnt pathway, since ROS themselves are able to regulate the transcriptional activity of β-catenin^54^. This may further contribute to the apoptosis inducing synergistic action of TS in the combination treatment since other PPARγ agonists have been demonstrated to increase ROS levels in lung cancer cells by altering glucose metabolism to reduce glutathione levels ultimately resulting in cell cycle arrest^36^. We have demonstrated an increase in caspase 3 activity in response to AD + TS treatment indicating apoptotic cell death, however active caspase 3 is also known to inhibit the Wnt pathway by cleaving β-catenin^55^. In addition TS dependent generation of ROS can upregulate expression of death receptors in lung cancer cells making them more susceptible to TNF-related apoptosis-inducing ligand (TRAIL) treatment^56^.

In sum, our results thus far have demonstrated that CSCs expand in lung cancer tumoroids and tumors wherein the Wnt signaling pathway plays a crucial role in lung CSC development and this signaling involves several important known targets such as Sox2, ALDH, and Nos2. Further, a combination treatment comprising two FDA-approved drugs, AD and TS, not only reduced the expression of stemness markers in lung cancer tumoroids *in vitro* but also was able to reduce tumor growth *in vivo*. Moreover, the pivotal role of Wnt signaling pathway was demonstrated in the synergism underlying the combination therapy.

## Methods

### Study design

The purpose of this study was to evaluate CSC expansion in NSCLC tumoroid cultures and to determine the effectiveness of AD + TS treatment in targeting CSCs. We evaluated CSC expansion in mouse and human tumoroids using multiple assays including qPCR, sphere formation, ALDH activity, and tumor initiation. Experiments were performed using a minimum of two biological replicates and three technical replicates. For the qPCR array two biological replicates were used and data was uploaded to Qiagen GeneGlobe data analysis center for fold change and p value calculations. The goal of the *in vivo* experiments was to determine the effectiveness of AD + TS in reducing tumor growth while minimizing the number of animals needed. Each animal experiment was performed as two independent studies with N for each group as described below. Mice were randomly assigned to treatment groups and tumor size was assayed by caliper measurement. Each experiment was terminated when the largest tumors reached 10 mm in diameter.

### Statistical analysis

Experiments have been repeated at least twice. Statistical significance for each experiment was determined using analysis of variance (ANOVA) and the Tukey post hoc test, p < 0.05. Calculations were performed, and graphs plotted using Prism 6.0 software (Graphpad Software, San Diego, CA, USA). Graphs of results show the mean and error bars depict the average ± standard deviation.

### Chemicals

AD was obtained from Acros Organics and TS was obtained from Selleck chem.

#### 3D Cell culture

Polymeric nanofiber scaffold was prepared as previously described^20^. Scaffolds were sterilized in ethanol and prepared for tumoroid culture as described^20^. Cell lines were obtained from ATCC. Cells were cultured in a humidified incubator at 37 °C in a 5% CO_2_ atmosphere (Thermo Fisher). 3D Tumoroid formation was assessed using fluorescent microscopy (Olympus BX51) after nuclear staining with Nuc Blue dye (Thermo Scientific).

#### Quantitative reverse transcriptase PCR

Total RNA was isolated from tissues or cell pellets using RNeasy columns (Qiagen). cDNA synthesis was performed using the Maxima cDNA synthesis kit (Thermo Scientific) according to manufacturer protocol. Real time analysis was performed using a Syber Green assay (Genecoepia) in a Bio Rad CFX-384 thermocycler using primers obtained from Integrated DNA Technologies (see Fig. S15 for sequences). Data analysis was performed in Bio Rad CFX Maestro software with a significance threshold of p ≤ 0.05.

#### Flow cytometry

All flow cytometry experiments were performed using a Becton Dickenson (BD) FACS Canto II system at the University of South Florida COM Fred Wright Jr. Flow Cytometry Core. Cell sorting was performed on a BD FACS Aria system at the Flow Cytometry Core at the H. Lee Moffitt Cancer Center & Research Institute.

#### Stem cell activity assays

ALDH assays were performed using the ALDE Flour kit (Stem Cell Technologies) according to manufactures instructions. Controls treated with Diethylamino-benzaldehyde (DAEB) were used to distinguish ALDH positive cells from background fluorescence.

### CSC quantification

CD24 expressing cells were depleted from A549 cell cultures using magnetic cell separation columns and CD24 antibody conjugated magnetic beads (Miltenyi Biotech) according to manufacturer’s instructions. Depletion was verified using flow cytometry as stated above. Antibody staining to determine CD44 and CD24 expression was performed using FITC conjugated anti-CD44 and Alexa 647 conjugated anti-CD24 from BD. Cells were stained and quantified by flow cytometry. Dead cells were identified and excluded using 4′,6-diamidino-2-phenylindole (DAPI) staining. Analysis was done in BD FACS Diva software.

#### IC_50_ Assay

IC_50_ of drug treatments in 3D scaffold or monolayer cultures were determined by Cell Titer Glo Assay (Promega). Culture media was changed to media containing drug dilutions on day 4 of culture and viability assay was performed after 48 hours on day 6 of culture. Cell Titer Glo reagent was added and incubated according to manufacturer’s instructions and luminescence was measured in a Bio-Tek Synergy H4 plate reader. Dose response curves were plotted and IC_50_ values were estimated using Graph Pad Prism software.

#### Sphere formation assay

Cells were plated at a density of 2 cells per µL in sphere media in low attachment plates (Corning) as previously described^57^, and drugs added 24 h post plating and spheres were imaged using brightfield microscopy after 6 d of culture. Sphere size and diameter was measured using imageJ software. Sphere efficiency is represented as the percentage of cells seeded that were able to proliferate under low attachment conditions and defined as the number of spheres counted at endpoint divided by the number of cells seeded times 100.

#### ß-catenin activity assay

Active *ß-catenin* was assayed using a Cignal TCF/ Luciferase reporter system (Qiagen). Briefly, cells were transfected with the control or reporter plasmid using Lipofectamine 3000 reagent on day 3 of culture (Thermo Scientific) and treated with drugs at stated concentrations 48 hours after transfection. Luciferase activity (firefly reporter and renilla transfection normalization control) was assayed using the Dual Glo luciferase assay (Promega) in a BioTek Synergy H4 plate reader after 48 hours of treatment.

#### Animal experiments

Animals were housed in the University of South Florida comparative medicine facility at the Morsani College of Medicine and all protocols were reviewed and approved by the USF Institutional Animal Care and Use Committee (IACUC). All experiments were performed in accordance with IACUC approved guidelines and regulations. C57BL/6 mice were purchased from Envigo and NSG immunocompromised mice (NOD.Cg-Prkdcscid Il2rgtm1Wjl/SzJ) were purchased from Jackson Laboratory. Subcutaneous tumors were grown in the flanks of mice by injecting one million LLC1 monolayer cells, 100,000 LLC1 tumoroids, or 5 million A549 cells. Tumors were allowed to grow and treatment was started when they became palpable (2–3 mm diameter by caliper). Drugs were injected intratumorally once every 3 days at the indicated concentrations. Drug solutions were made in PBS with 1% DMSO and this solution was used as vehicle control. Tumors were collected when controls reached 10 mm in diameter. Tumor volume was estimated by the formula V = ((W^2^ * L)/2). To obtain single cell suspensions of tumors the tissue was digested using a mouse tumor digestion kit and Gentle MACS instrument (Miltenyi Biotech) according to manufacturer instructions.

#### PCR Array

Gene expression analysis was performed on LLC1 3D cultures using the cancer stem cell RT^2^ PCR profiler array (Qiagen). RNA was isolated as stated above and processed according to manufacturer instructions. Array plates were read in a Bio Rad CFX-384 thermocycler and analysis was performed using the Qiagen Gene Globe Data Analysis Center web application.

#### Protein Abundance Immunoassays

Immunoassays for relative protein abundance were performed using Wes according to manufacture instructions (Protein Simple). Total protein was isolated using RIPA buffer and protein concentration was determined by Bradford assay. Antibodies used in this assay are: Caspase3 (Cell Signaling #9662p) PARP (Cell Signaling #9542) β-actin (Sigma #A2228).

#### Pathway network analysis

Ingenuity Pathway Analysis (IPA, QIAGEN Inc., https://www.qiagenbioinformatics.com/products/ingenuity-pathway-analysis) was used to identify the pathways that are known to be significantly affected by treatment with AD and TS.

#### Wnt pathway phospho-antibody array

Tumors from each treatment group were snap frozen on dry ice and stored at −80 °C. Protein was isolated using an antibody array assay kit (Full Moon Biosystems) and loaded onto Wnt phospho explorer array slides (Full Moon Biosystems) and analyzed as per manufacturer instructions.

#### ICC

Tumoroids were fixed and permeabilized on scaffold for 15 minutes using 4% paraformaldehyde/0.05% tween 20 solutions and blocked for 15 minutes in 10% FBS prior to staining with Nos2 antibody (Cell Signaling #D6B6S) and anti-rabbit Alexa 488 secondary (Life Technologies).

#### Assay for the production of ROS

For assessment of ROS, cells were stained in PBS containing 5 µM CM-H2DCFDA at 37 °C for 20 minutes. Following staining cells were washed in PBS. Staining was assayed immediately using fluorescence microscopy with a GFP filter. Brightfield images were also acquired for reference. Staining was also assayed by flow cytometry (BD FACS Canto/Diva).

## Supplementary information

Supplementary File
