# Supplementary material for: Actinomycin D and Telmisartan Combination Targets Lung Cancer Stem Cells Through the Wnt/Beta Catenin Pathway
Source: Sci Rep. 2019 Dec 3;9:18177. doi: 10.1038/s41598-019-54266-z (PMC6890794; doi:10.1038/s41598-019-54266-z)
Supplement: Supplementary file 1 — Supplementary File [file 41598_2019_54266_MOESM1_ESM.pdf]

Actinomycin D and Telmisartan Combination Targets Lung Cancer Stem Cells Through the Wnt/Beta Catenin Pathway

Ryan Green, Mark Howell, Roukiah Khalil, Rajesh Nair, Jiyu Yan, Elspeth Foran, Sandhyabanu Katiri, Jit Banerjee, Mandip Singh, Srinivas Bharadwaj, Shyam S Mohapatra and Subhra Mohapatra

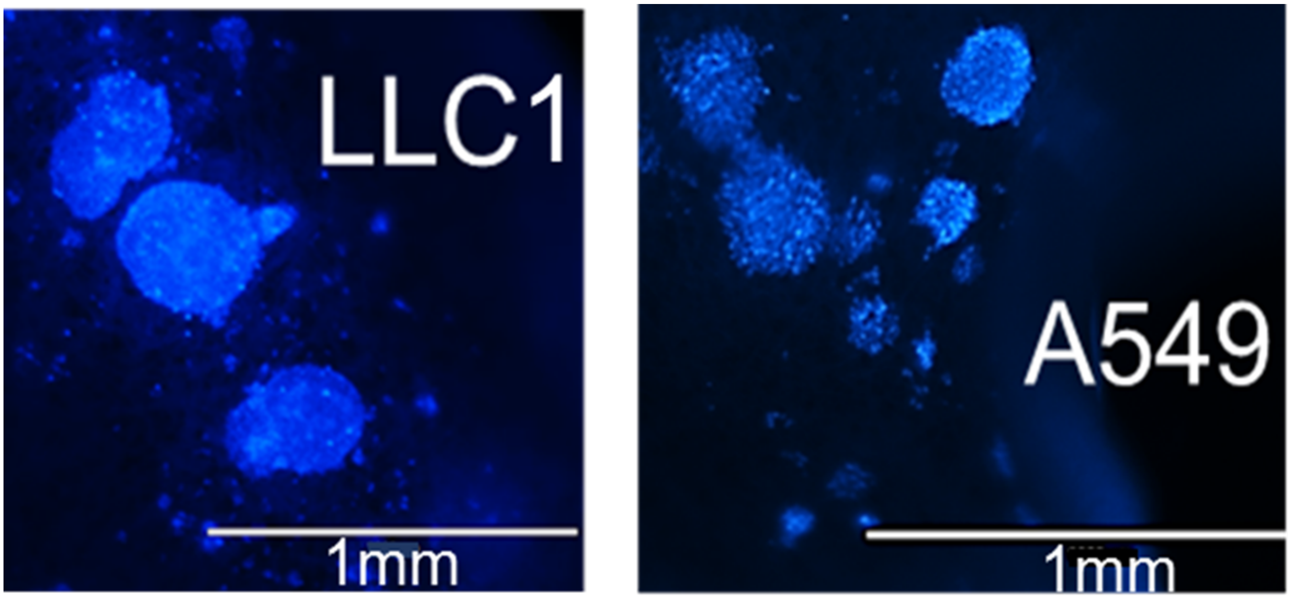

Figure S1. Enlarged micrograph from figure 1A.

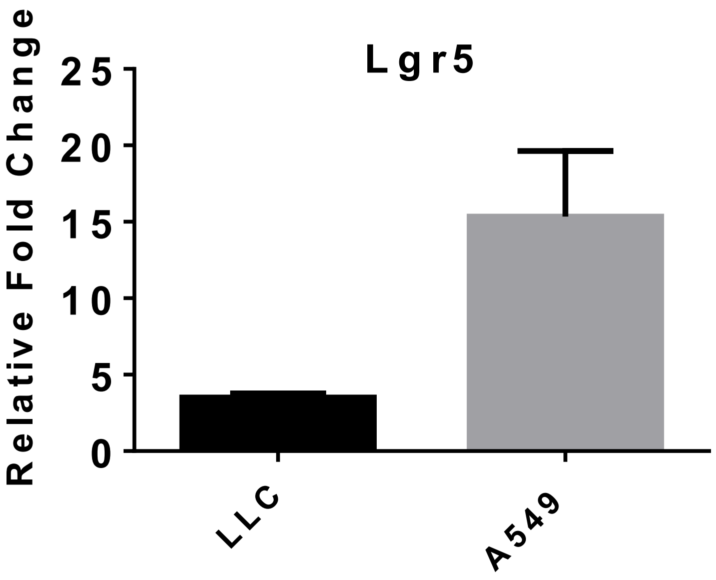

Figure S2. Lgr5 mRNA expression. Lgr5 expression was assayed in LLC and A549 scaffold cultures and presented as fold change relative to the respective monolayer culture.

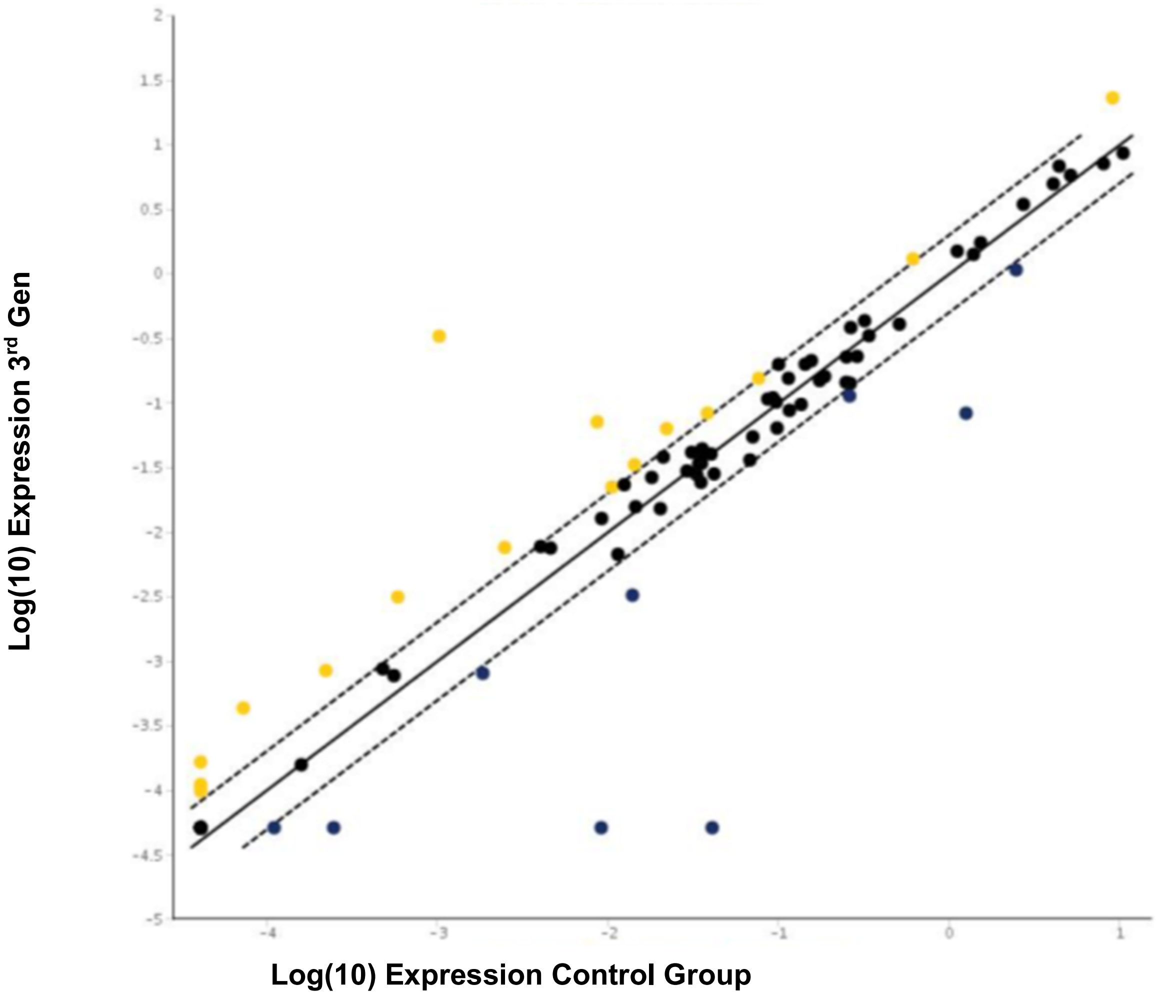

Figure S3. CSC qPCR Array Scatter plot depicting gene expression changes in LLC1 monolayer vs 3rd generation LLC1 scaffold observed in the cancer stem cell qPCR array (Qiagen Gene Globe Analysis Center).

DRUG NAME	IC50 (μM)
**Actinomycin-D**	**0.03**
Mitomycin	0.05562
Plicamycin	0.2801
Daunorubicin HCL	0.3407
4'-Epiadriamycin	0.3661
Doxorubicin HCL	0.4195
bortezomib	0.937
Mechlorethamine hydrochloride	1.127
mitozantrom	1.44
topotican	2.19
Gemcitabine	2.525
Depsippeptide	4.727
Thioguanine	6.913
4-DMD	7.58
Etoposide	8.81
Carfilzomib	10.06
sunitinib	10.38
methotrexate	11.34
Pazpanib	11.97
sorafenib tosylate	12.78
PF2341066	13.66
BIBW2992	15.9
Oxaliplatin	17.1

Figure S4. Drug screening with NCI Diversity set in lung cancer cell lines. Cell viability assayed using CellTiter-Glo and IC50 calculated using Graph pad prism software.

Tumoroids AD+TS Combination Index

LLC

Dose AD(nM)	Dose TS(nM)	CI
200.0	1000.0	0.08389
100.0	1000.0	0.32866
50.0	1000.0	0.37834
25.0	1000.0	0.34917

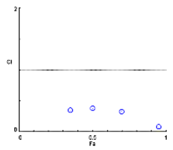

H1299

Dose AD(nM)	Dose TS(nM)	CI
0.4	1000	2.49798
1.2	1000	1.94224
3.7	1000	1.38684
11.1	1000	1.57374
33.33	1000	0.08410
100.0	1000	0.03756

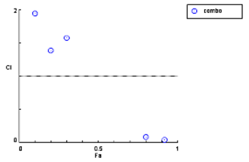

A549

Dose AD(nM)	Dose TS(nM)	CI
0.4	1000.0	0.30436
1.2	1000.0	0.26114
3.7	1000.0	0.63686
11.1	1000.0	0.90548

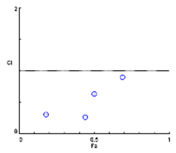

H460

Dose AD (nM)	Dose TS (nM)	CI
100.0	1000	2.08297
33.33	1000	0.32465
11.0	1000	0.35910
3.7	1000	1.67062
1.2	1000	0.82078
0.4	1000	0.43460

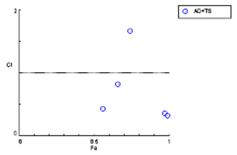

CI ≤ 1 = Synergy

Figure S5. Combination Index for AD+TS Combination index was used to determine synergy between TS (10μM) and AD (varying doses). CI was calculated from viability data using CompuSyn software.

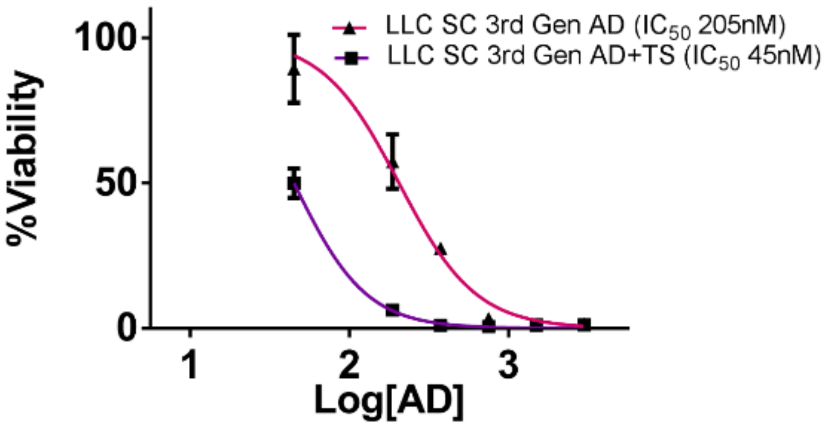

Figure S6. AD+TS Synergy in 3rd Generation tumoroids. The IC50 values for AD and AD+TS were determined in 3rd generation scaffold LLC1 after 48hr treatment with increasing concentrations of AD and/or 10μM TS. Cell viability was determined using CellTiter-Glo assay.

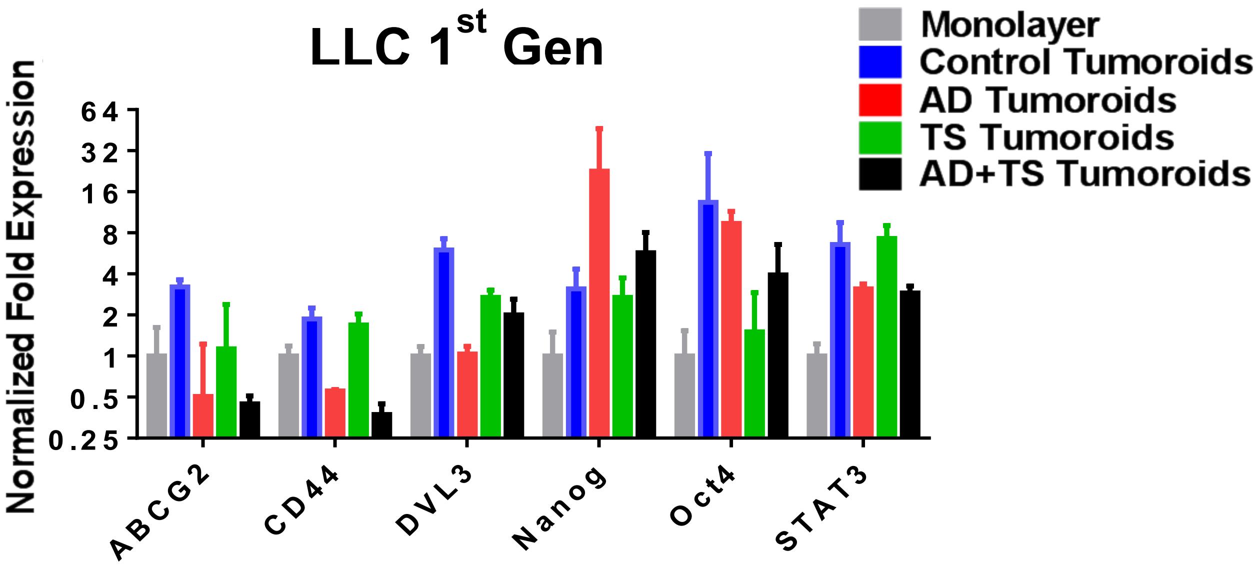

Figure S7. mRNA expression in LLC 1st generation tumoroids following 24hr drug treatment normalized to LLC monolayer. Drugs were added on day 4 of culture and cells were collected on day 5. Expression of CSC related genes were assayed by qPCR

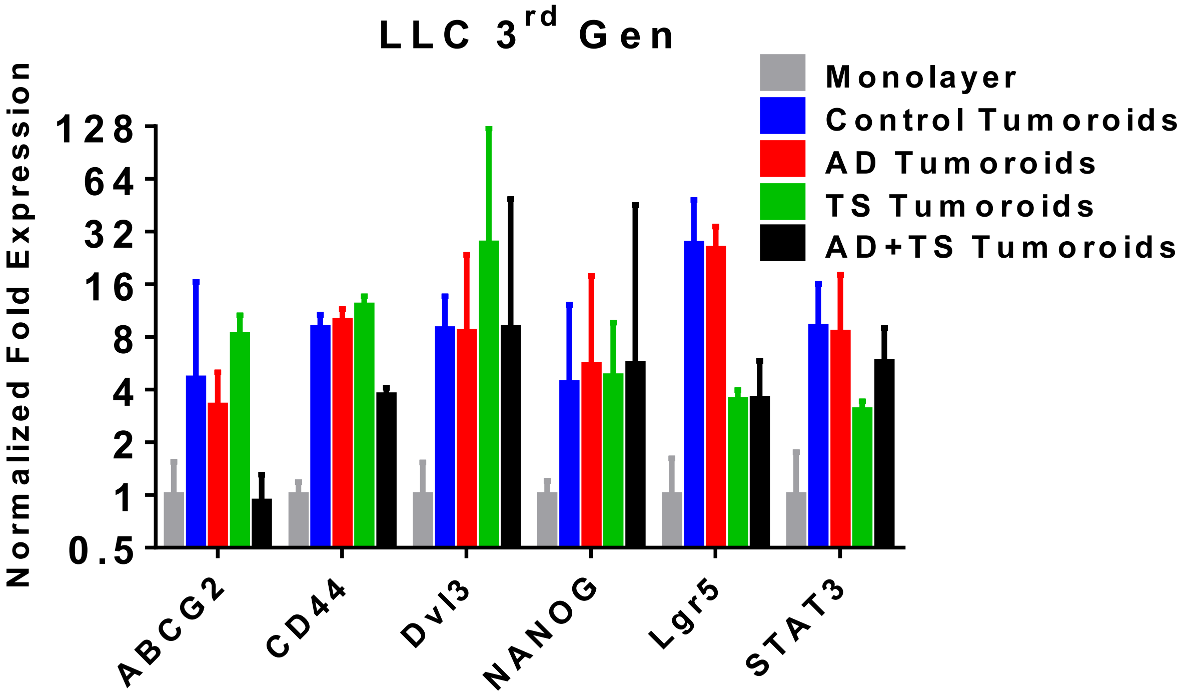

Figure S8. mRNA expression changes in LLC 3rd generation tumoroids with or without drug treatment normalized to LLC monolayer. Drugs were added on day 4 of culture and cells were collected on day 6. Expression of CSC related genes were assayed by qPCR.

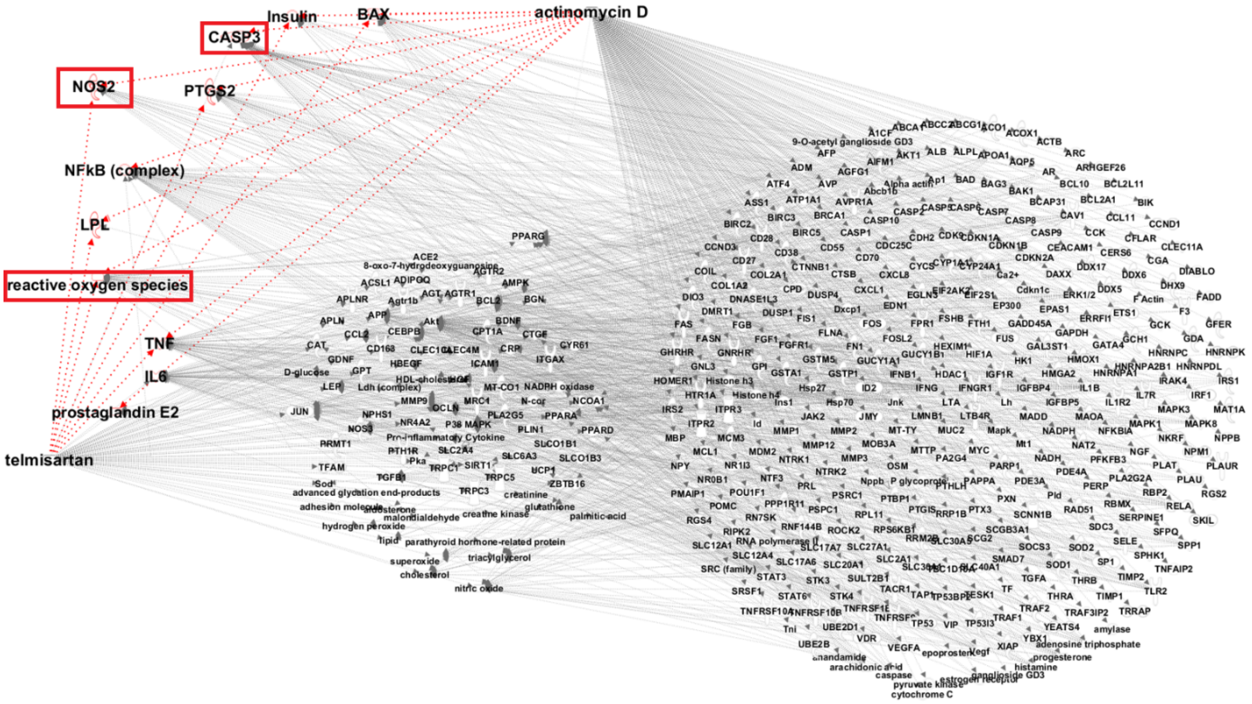

Figure S9. Ingenuity Pathway Analysis. Data were analyzed through the use of IPA (QIAGEN Inc., https://www.qiagenbioinformatics.com/products/ingenuity-pathway-analysis) to find molecules that are affected by both AD and TS. Direct interactions are highlighted in red, indirect interactions are shown in grey.

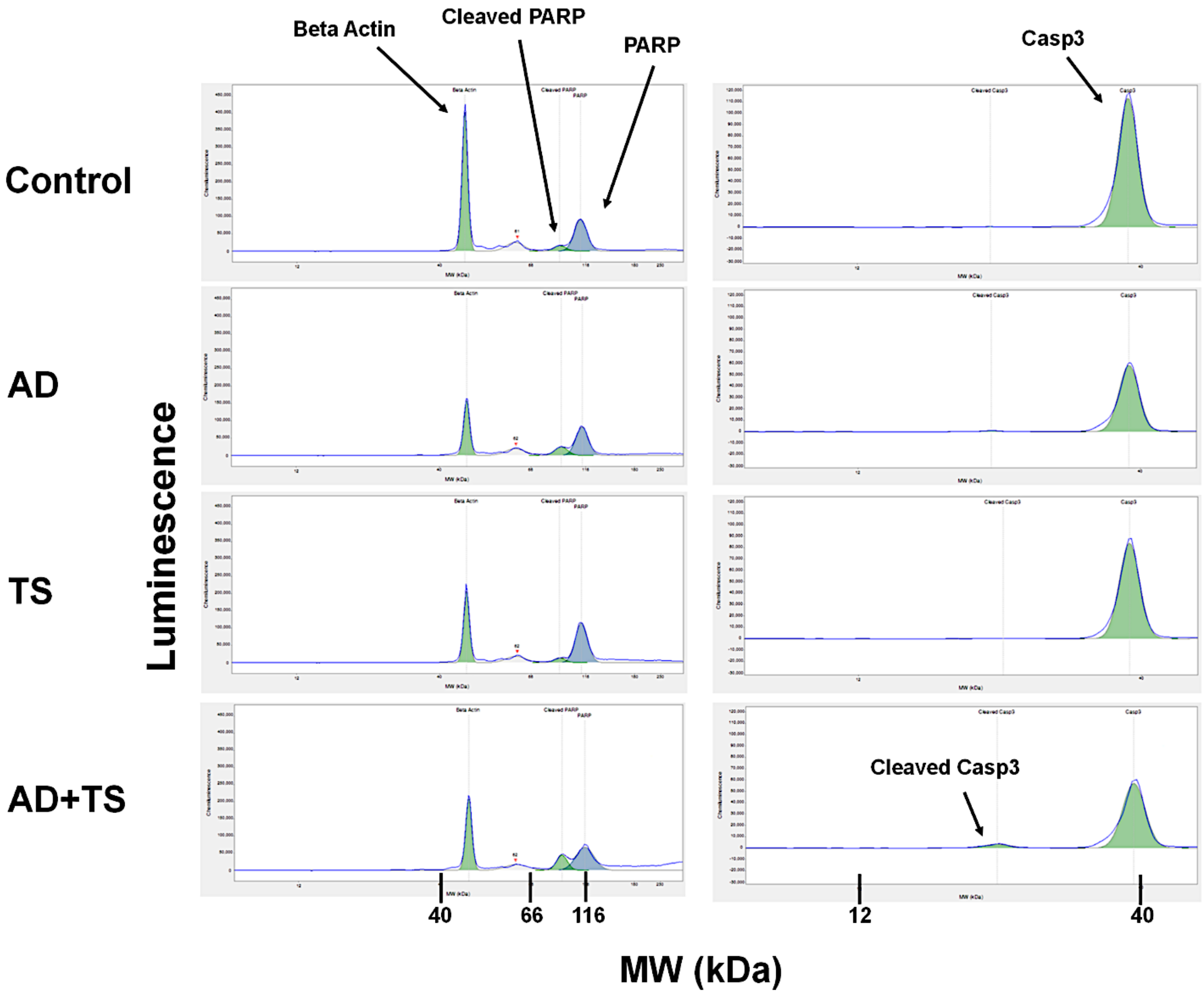

Figure S10. Western Immunoassay Luminescence Peaks Luminescence values and corresponding molecular weights obtained using Wes for PARP, Caspase3 and Beta actin for the experiment presented in figure 4

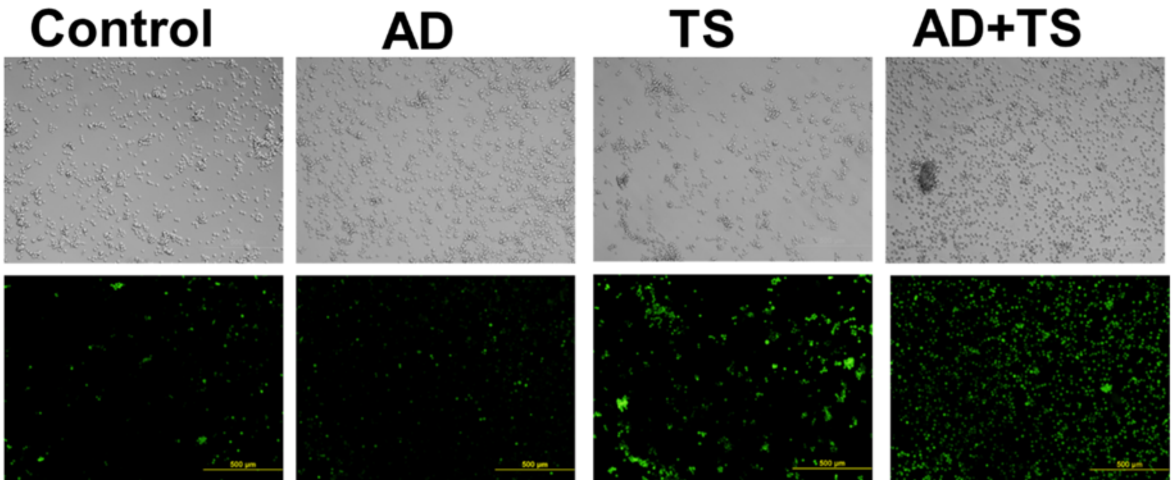

Figure S11. ROS Staining Fluorescent and bright field microscopy images following drug treatment and CM-H2DCFDA staining as described in figure 4F.

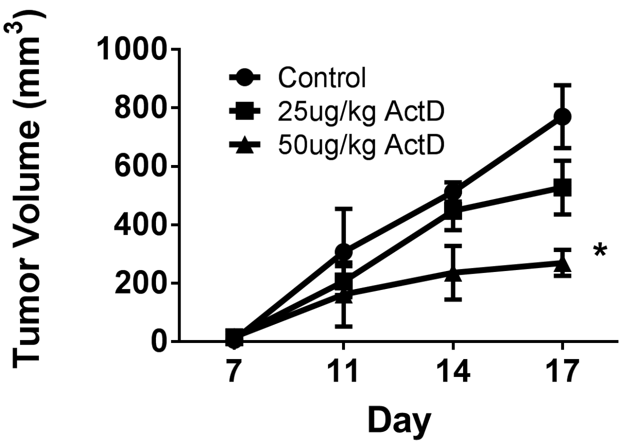

Figure S12. LLC Tumor Response to AD Tumor growth initiated by 1 million LLC1 monolayer cells during treatment with either 25 or 50 µg/kg AD.

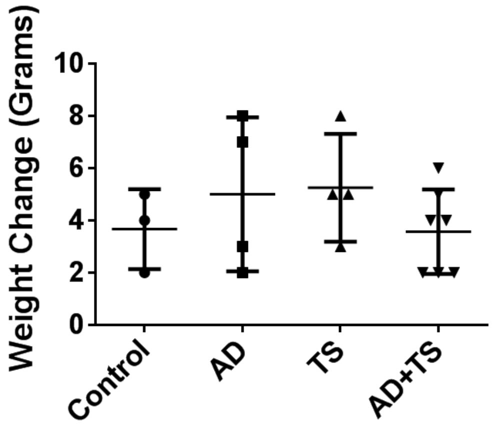

Figure S13. Mouse weight change between day of first drug treatment to endpoint corresponding to figure 5C.

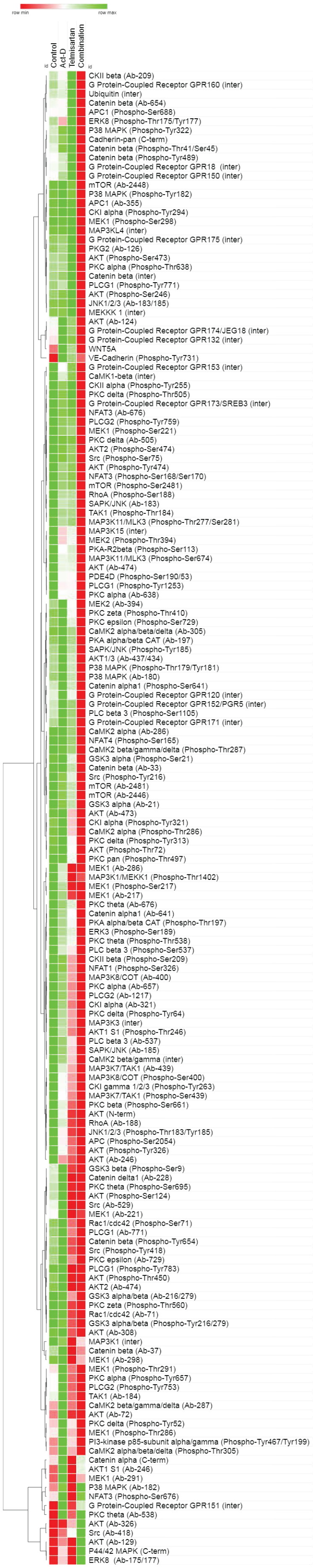

Figure S14. Wnt Pathway Phospho Array. Heatmap Depicting relative abundance of proteins involved in the Wnt pathway in drug treated LLC1 tumors. Heatmap depicting relative protein abundance and phosphorylation for Wnt pathway proteins in drug treated LLC1 tumors. Protein was collected from drug treated LLC1 tumors (Figure 5B) and a phospho antibody array assay was performed to determine changes occurring in the Wnt pathway (Full Moon Biosystems). Cy-3 developed array chips were sent to Full Moon Biosystems for scanning and images were analyzed using ImageJ to determine relative normalized staining intensity for each antibody.

Mouse

Gene	Primer Sequence
B-Actin	GGGGTGTTGAAGGTCTCAAA<br>AAATCTGGCACCACACCTTC
STAT3	AGAAAGCTGGGTCACATGGGGGAGGTAGCACAC<br>AAAAAGCAGGCTCCAGGATGGCTCAGTGGAACCAG
ALDH1a1	GGCCACACACTCCAATAGGT<br>GCACTCAATGGTGGGAAAGT
NANOG	ACTGGTAGAAGAATCAGGGCT<br>TTGCTTACAAGGGTCTGCTACT
Oct4	GCTTAGCCAGGTTCGAGGAT<br>TAGGTGAGCCGTCTTTCCAC
Sox2	GAAGCGCCTAACGTACCACT<br>TTAACGCAAAAACCGTGATG
c-myc	GCCAAGGTTGTGAGGTTAGG<br>TAACTCGAGGAGGAGCTGGA
Dvl2	GCGCTGGATACTGGTAGGAG<br>TGACAATGACGGTTCCAGTG
Dvl3	AAAAGGCCGACTGATGGAGAT<br>AGGGCCCCTGTCCAGCT
Nos2	GCCCCTCACCATTATCTTTAC<br>TGTGGCTACCACATTGAAGA
CD44	AAATGCACCATTTCCTGAGA<br>AGAAGGTGTGGGCAGAAGAA
ABCG2	CAGGGCCACATGATTCTTCC<br>TCTGTCTTCCTGGTCCTCTC

Human

Gene	Primer Sequence
B-Actin	GGGGTGTTGAAGGTCTCAAA<br>TTCTACAATGAGCTGCGTGTG
Nanog	CTCGCTGATTAGGCTCCAAC<br>CAGTCTGGACACTGGCTGAA
OCT4	TCCAGGTTTTCTTTCCCTAGC<br>TGTACTCCTCGGTCCCTTTC
SOX2	GCAAGAAGCCTCTCCTTGAA<br>GCTAGTCTCCAAGCGACGAA
CD44	GGTGATCCAGGGACTGTCTT<br>AGACATCTACCCCAGCAACC
ALDH1a1	CACGGGCCTCCTCCACATT<br>AGGGGCAGCCATTTCTTCTCA
STAT3	TGAAGCTGACCCAGGTAGCGCTGC<br>GCCAGAGAGCCAGGAGCA
c-myc	CTCAGCCAAGGTTGTGAGGT<br>CGGAACTCTTGTGCGTAAGG
Dvl2	AACCTGGATAGGCTGGGAAG<br>TGAGCAACGATGACGCTGTG
Nos2	GCAGGTCACTTATGTCACTTATC<br>GTTCTCAAGGCACAGGTCTC
ABCG2	TACGACTGTGACAATGATCTGAGC<br>TTTCCAAGCGTTCATTCAAAAA
Plat	GAGAAGTACAGGGCCTGCTG<br>GAAGAGAGGGCTCTGCTGTG
uPar	CTACAGCGCTGACACGCTTG<br>TGCGTCCTGGTCGTGAGCGA
Dvl3	TGTGCGAGGTTTAAGGTCTA<br>AGGTGCCTATGCAAGTTCA
ABCG2	TTTCCAAGCGTTCATTCAAAA A<br>TACGACTGTGACAATGATCTGAGC

Figure S15. Primer sequences used for quantitative real time PCR.
